# Associations between Feeding Patterns and Infant Health in China: A Propensity Score Matching Approach

**DOI:** 10.3390/nu13124518

**Published:** 2021-12-17

**Authors:** Yuehui Fang, Yiyao Lian, Zhenyu Yang, Yifan Duan, Yuna He

**Affiliations:** Key Laboratory of Trace Element Nutrition of National Health and Family Planning Commission, National Institute for Nutrition and Health, Chinese Center for Disease Control and Prevention, No. 29 Nanwei Road, Xicheng District, Beijing 100050, China; fangyh@ninh.chinacdc.cn (Y.F.); lianyy@ninh.chinacdc.cn (Y.L.); yangzy@ninh.chinacdc.cn (Z.Y.); duanyf@ninh.chinacdc.cn (Y.D.)

**Keywords:** infant, feeding pattern, cross-sectional study, propensity score matching

## Abstract

Breastmilk is the optimal food for infants. Feeding pattern is closely related to physical development and health during infancy. Understanding the associations between feeding patterns and health status can inform related policy interventions and advocacy in China. This study aimed to investigate the relationship between infant feeding patterns and health status in China infants. The China National Nutrition and Health Surveillance 2013 was a national-representative cross-sectional study performed particularly for children aged 0–5 years. A total of 3974 infants aged under 1 year were included in the analysis, of whom 1082 (27.2%) made up the formula feeding group, and 2892 (72.8%) made up the breastfeeding group. The associations between feeding patterns and physical development and health were investigated using propensity score matching and multivariable logistic regression models. Among breastfeeding and formula feeding infants aged 9–11 months old, weight-for-age z score was 1.1 ± 1.1 and 0.9 ± 1.3, respectively, and weight-for-length z score was 1.0 ± 1.3 and 0.7 ± 1.4, respectively. Hemoglobin in 0–2, 3–5, 6–8, and 9–11 months old breastfeeding infants was 121.4 ± 15.2 g/L, 117.1 ± 13.0 g/L, 113. 9 ± 11.9 g/L, and 114.4 ± 14.0 g/L, while in 0–2, 3–5, 6–8, and 9–11 months formula feeding infants was 116.3 ± 14.8 g/L, 120.4 ± 11.3 g/L, 119.8 ± 11.2 g/L, and 120.0 ± 11.5 g/L, respectively. Breastfeeding was associated with lower risk of respiratory disease (OR: 0.79; 95% CI: 0.64, 0.99) and diarrhea (OR: 0.75; 95% CI: 0.57, 0.98). Breastfeeding could slightly improve infant physical development, and had a protective effect on the diarrheal and respiratory diseases. Infants aged 3–11 months who were breastfeeding showed lower hemoglobin than that of formula-fed infants and thus should increase intake of iron rich complementary foods.

## 1. Introduction

Early nutrition is critical for the development of children. Human milk is the optimal food for infants and is closely related to health during infancy. Early and prolonged breastfeeding has been proven to ensure the best possible health and developmental outcomes for the infant [1]. Exclusive breastfeeding for four to six months is generally considered one of the best protective measures to prevent allergy and associated illnesses [2]. According to previous studies, breastfeeding is protective against acute respiratory infection and diarrhea [3,4,5], and is considered to be one path to lower healthcare economic costs due to lower incidence of diseases in both breastfed infants and breastfeeding mothers [6]. Breast milk is rich in antimicrobial substances that are important in preventing early-life infections, including immunoglobulins, complement proteins, lysozyme, lactoferrins, and oligosaccharides [7,8]. Moreover, human milk consumption introduces the infant to highly diverse and complex bacterial communities [9]. The World Health Organization (WHO) and Chinese dietary guidelines both recommend that children be exclusively breastfed for six months, and continue to be breastfed until two years of age or beyond [10].

The majority of people’s lifestyles have changed dramatically in both rural and urban areas as a result of China’s process of urbanization. The feeding patterns of infants under the age of 12 months have changed greatly. Since the 1990s, China has made numerous efforts to encourage breastfeeding [11]. However, between 1985 and 2015, in China’s urban area, the formula feeding rate among infants under 6 months and the continuous breastfeeding rate at 1 year of age were remained stable at around 15% and 30%, respectively, while in the suburbs, the formula feeding rates increased from 6.5% to 15.2% and continuous breastfeeding rates decreased from 60.1% to 29.8% [12]. According to the most recent results of the Chinese nutrition and health survey in 2013, exclusive breastfeeding rate among infants aged 0–6 months was 18.6% [13].

Feeding pattern is one of the environmental determinants related to weight gain and subsequent obesity [14]. Breastfeeding has been regarded as associated with lower weight and length gain throughout infancy when compared with formula feeding [15]. Formula-fed infants, on the other hand, are believed to be more prone to acquire weight and develop out of proportion to linear growth [16]. Breastfeeding is also thought to play a role in preventing obesity in infants and non-communicable diseases in adults [17]. Nevertheless, some studies yielded contradictory results. A recent study reported that the amount of formula was unrelated to weight [18]. A systematic review showed that an inverse association between exclusive breastfeeding duration and weight and length gain during infancy was only found in the developed setting [19]. Another systematic review suggested that breastfeeding may protect against being overweight or obese, but residual confounding cannot be ruled out [20].

As far as we know, except for feeding practice, biomedical and behavioral factors could also influence physical and immune system development during infancy [21], including preterm delivery [22], low birth weight (LBW) and intrauterine growth restriction [23], infectious diseases [24], sleep duration [25,26], physical activities [27], and complementary food feeding [28,29]. Infant development may be influenced by sociodemographic and maternal factors such as maternal gestational weight gain [30], gestational age, employment and education, family income and regional economic level [24,25,26,27,28,29,30,31] could also affect infant development. These variables were also related to infants’ feeding patterns [32]. These confounding factors may have an impact on the results of infant feeding studies.

In light of the above, it is critical to account for confounding factors when assessing the relationship between feeding patterns and physical and heath health in infants, which necessitates the use of proper study methods. Knowing the association between feeding patterns and physical health in Chinese infants is essential to health practitioners and policymakers in China and will help to promote infant health.

## 2. Materials and Methods

### 2.1. Data Sources

**Figure 1:**
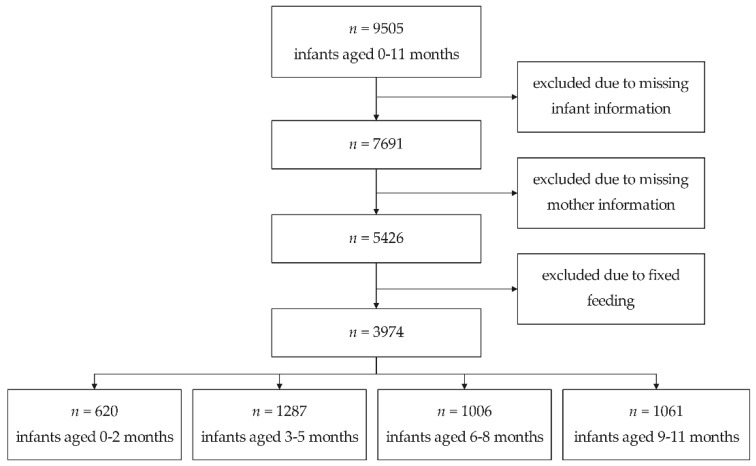
Flowchart of study participants selection.

Data for this study was obtained from the 2013 China National Nutrition and Health Survey (CNNHS). The China National Nutrition and Health Surveillance 2010–2013 was a national-representative cross-sectional study that covered 30 provinces. It was conducted by the National Institute of Nutrition and Health, Chinese Center for Disease Control and Prevention (NINH, Chinese Center for Disease Control and Prevention). All of the participants in this study were selected during the surveillance round 2013, which targeted children aged 0 to 5 and lactating mothers. The surveillance used stratified proportional random cluster sampling methods to ensure it had national representatives. As described in previous study, a total of 2865 districts/counties in China were categorized into four strata (large cities, medium and small cities, general rural areas, and poor rural areas), from which 55 counties (12 metropolis, 15 medium and small cities, 18 general rural areas, and 10 poor rural areas) were chosen in the study. In each selected county, three communities/townships were systematically sampled. In each selected township, three neighborhood/villages were systematically selected. Finally, 10 children from each age group were randomly selected in each selected village [13]. All participants and their guardians were required to complete an informed consent form before participating in the study. The Ethics Review Board of the Institute for Nutrition and Health, China CDC approved the protocol (2013–018). Only infants and their mothers both with complete basic and diet information were included in the analysis. Separate analyses were carried out for infants aged 0–2 months, 3–5 months, 6–8 months, and 9–11 months (Figure 1).

### 2.2. Outcome Variables

The outcome variables were body length, body weight, weight-for-age z score, length-for-age z score, weight-for-length z score, hemoglobin, and prevalence of respiratory disease and diarrheal disease. Z-scores were calculated based on the WHO Child Growth Standards based on length/height, weight, and age [33]. Respiratory disease and diarrheal disease were measured based on maternal recall of symptoms of cough and shortness of breath, and diarrhea during the last two weeks, respectively. Blood samples were collected from infants’ finger using capillary blood extraction. Cyanide high-iron method was used to determine hemoglobin concentration.

### 2.3. Exposure Variables

We investigated infants’ intake of breast milk, formula, and complementary food during the previous week. Breastfeeding data were collected by recall interviews carried out face-to-face with the parents or caregivers of the infants. Feeding patterns were classified into two types for infants under 1 year of age based on their sources of milk.

- Breastfeeding infants were those whose source of milk was only breast milk (inclusive of mother’s own milk by bottle);
- Formula feeding were those whose source of milk was only formula.

Because the proportion of feedings containing breast milk is unknown, fixed feeding was not included in the analysis.

### 2.4. Potential Confounders

The potential confounders were selected based on the results of previously published studies [21,22,23,24,25,26,27,28,29,30,31] and the availability of data. Potential confounding factors were broadly classified into socioeconomic and demographic factors, biomedical and behavioral factors, and maternal factors.

Socioeconomic and demographic factors included place of usual residence, household wealth status, and mother’s education and occupation. Based on the socio-economic level of the survey sites, places of usual residence were classified as big cities, medium and small cities, normal rural, and poor rural. Annual household income per capita was classified into three categories: low (CNY 10,000), medium (CNY 10,000–19,999), and high (CNY 20,000) to describe household wealth status. Mother’s education was categorized into four categories: elementary school or below, junior school, high school, high school, and college and above. Mother’s occupation was categorized into three categories: professionals, working in the agricultural industry, and others.

Infant biomedical and behavioral factors included gestational age, birth weight, physical activity, sleep duration, and complementary food intake. Gestational age was divided into two groups: preterm infants were defined as gestational age < 37 weeks, term infants were defined as gestational age ≥ 37 weeks. Birth weight was divided into three groups. Low birth weight (LBW) was defined as birth weight < 2500 g, whereas macrosomia was defined as birth weight > 4000 g. According to Guidelines on physical activity, sedentary behavior, and sleep for children under 5 years of age [34], the continuous variable reflecting hours of sleep duration was dichotomized into sufficient (sleep duration ≥ 14 h/day for infant younger than 3 months, sleep duration ≥ 12 h/day for infant above 3 months) and insufficient (sleep duration < 14 h/day for infant younger than 3 months, sleep time < 12 h/day for infant above 3 months), and physical activity time was dichotomized into high (activity time ≥ 30 min/day) and low (activity time < 30 min/day). For infants over the age of 6 months, complementary food was matched to the types of complementary foods consumed in the previous week, which included grains, tubers, vegetables, fruits, legumes, eggs, meat, aquatic products, and dairy.

Maternal factors included maternal age and gestational weight gain. Maternal age was categorized into three categories: <20 years, 25–35 years, and >35 years. Gestational weight gain was categorized into insufficient, appropriate, and excessive according to the Institute of Medicine (IOM) [35].

### 2.5. Statistical Analysis

Data were entered through a standardized data management platform and were cleaned for all variables. Normally distributed data were assessed using the paired *t*-test, whereas for non-normally distributed data, a paired Wilcoxon rank-sum test was applied. A chi-square test was used for categorical variable comparison.

Propensity score matching (PSM) analysis was performed to reduce potential selection bias with the potential confounders. Even though cross-sectional surveys are useful for examining the relationships between exposure and outcome variables, unlike randomized controlled trials (RCTs), non-randomized self-selection in the exposure could confound the measure of association between the exposures and the outcomes [36]. Rosenbum and Rubin [37] proposed the PSM method to minimize the imbalance in participant characteristics between exposed and unexposed groups by considering the confounding between the two groups. PSM is a method for balancing the propensity scores of the exposed and unexposed groups so that covariate comparisons between the two groups can be made directly.

Multivariable logistic regression was used to investigate the associations between feeding patterns and respiratory and diarrheal diseases. Odds ratios (ORs) with 95% confidence intervals (CIs) were reported as the measure of association between feeding patterns and diseases. Data clean and analysis were performed using SAS 9.4 software (SAS Institute Inc., Cary, NC, USA). Propensity scoring and matching were conducted using the MatchIt package (version 4.1.0) for R software (R for Windows 4.0.5).

## 3. Results

The general characteristics before and after matching were described in Table 1. A total of 3974 infants were included in the analysis, of whom 1082 (27.2%) made up the formula feeding group, 2892 (72.8%) made up the breastfeeding group. Of the total infants, 2226 (56.0%) were from urban areas and 1748 (44.0%) were from rural areas, 2014 were boys (50.7%) and 1960 were girls (49.3%). Breastfeeding infants made up nearly three times the number of formula-fed infants. Between two feeding groups, there were differences in living area, annual income per capita, whether the infant was born to term or not, birth weight, physical activity time, complementary food intake, and mother’s educational level and occupation. After propensity score matching, no significant differences were found between two groups for the matched covariates.

**Figure 2:**
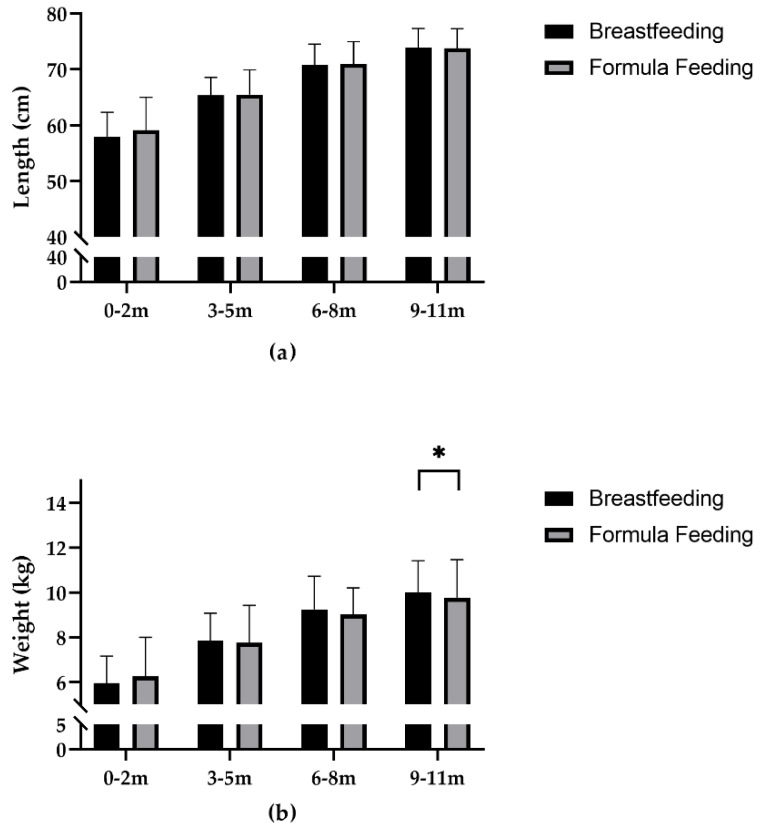

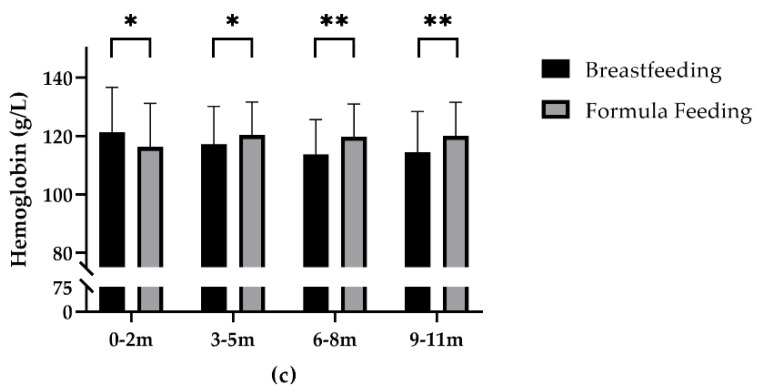
(**a**) Body length in breastfeeding and formula feeding 0–2, 3–5, 6–8 and 9–11 month infants was 58.0 ± 4.4 vs. 59.1 ± 5.9, 65.4 ± 3.2 vs. 65.5 ± 4.5, 70.8 ± 3.7 vs. 71.0 ± 4.0, and 73.8 ± 3.5 vs. 73.8 ± 3.5, respectively; (**b**) body weight in breastfeeding and formula feeding 0–2, 3–5, 6–8 and 9–11 month infants was 6.0 ± 1.2 vs. 6.3 ± 1.8, 7.9 ± 1.2 vs. 7.8 ± 1.7, 9.3 ± 1.5 vs. 9.0 ± 1.2, and 10.0 ± 1.4 vs. 9.8 ± 1.7, respectively; (**c**) hemoglobin in breastfeeding and formula feeding 0–2, 3–5, 6–8 and 9–11month infants was 121.4 ± 15.2 vs. 116.3 ± 14.8, 117.1 ± 13.0 vs. 120.4 ± 11.3, 113.9 ± 11.9 vs. 119.8 ± 11.2, and 114.4 ± 14.0 vs. 120.0 ± 11.5, respectively; * *p* < 0.05; ** *p* < 0.01.

In all age groups, there was little difference in infant body length between the breastfeeding and formula feeding groups. The difference of body weight between the two groups was minimal among infants under 6 months of age. However, among infants aged 6 to 11 months, body weight, weight-for-age z score and weight-for-length z score were significantly higher in breastfeeding group than those in formula feeding group. With the exception of the 0–2 months group, hemoglobin in the breastfeeding group was significantly lower compared with the formula feeding group (Table 2, Figure 2).

The prevalence of respiratory and diarrheal diseases during the last two weeks in breastfeeding group was lower than that in the formula feeding group (Table 3). Breastfeeding was found to have a significant protective effect against respiratory diseases in infants aged 3–5 months. Infants aged 6-8 months showed a significant protective effect against diarrhea. However, the protective effect on diarrhea among 0–2 months old infants was nearly reached statistical significance (*p* = 0.054).

## 4. Discussion

By using the national representative data, we described the status of breast and formula feeding in different sociodemographic population in China. Breastfeeding rates among infants under 1 year of age have been declining as they have grown older. Breastfeeding had a positive effect on weight gain in infants aged 6 months and up, according to our findings. However, no significant association has been observed between feeding pattern and body length gain in infants under the age of one. When compared to formula feeding, breastfeeding was linked to a lower risk of diarrhea and respiratory diseases. Despite these benefits, breastfeeding was associated with a lower hemoglobin level in infants older than 3 months.

Consistent with previous studies [24,25,26,27,28,29,30,31,32], we found that sociodemographic, biomedical, behavioral, and maternal factors, such as family income and regional economic status, as well as maternal employment and education, were all strongly related to the infant feeding patterns. During the process of urbanization in China, the maternal employment rate has risen dramatically. Several studies have indicated that the duration of maternity leave has a significant impact on the sustainability of breastfeeding among female workers [38,39]. Our study found that infants under 3 months of age were mostly (91.70%) breastfed, while only half of the infants older than 9 months were breastfed. Breastfeeding rates in big cities, medium and small cities, normal rural areas, and poor rural areas were 68.8%, 68.2%, 82.0%, and 81.0%, respectively. With the increasing annual income per capita, the breastfeeding rate decreased from 78.5% to 69.4%. These results might be attributed to the employment of mothers. Previous studies had demonstrated that employed mothers were more likely to have lower breastfeeding rate than unemployed mothers [40,41,42,43]. In China, the legal maternity leave is 98 days, and can be extended up to seven months by the employers. This might explain the phenomenon of a high breastfeeding rate under 3 months compared to a low breastfeeding rate after 9 months. The childbearing age of mothers could be another factor affecting infant feeding practice. Previous studies have shown that older mothers are more likely to breastfeed, but this study found that the breastfeeding rates decreased as mothers’ ages increased. This might be related to the socio-economic status. Mothers with a maternal age of older than 35 years might be more likely to have a better economic status and a higher work participation, making it more difficult to breastfeed. As a result, national policies are critical in reducing the conflict between employment and sustained breastfeeding [39].

Breast milk is optimal and essential for infant growth and development, and provides a rich source of early nutrition for infant growth and development [44]. Consistent with previous study [45], our study suggested that weight and length gain showed no significant statistical difference among formula-fed infants and breastfed infants for infants aged under 6 months. As for infants 6–11 months, we found that breastfeeding had some benefit on weight gain. According to a previous study, even if breastfeeding infants have a higher weight and BMI than bottle feeding infants, breastfeeding was still associated with a reduction in the odds of overweight and obesity [46]. On the other hand, the proportion of energy from complementary foods largely increased among infants aged 9–11 months, which may present addition confounding factors influencing physical development. Nevertheless, these results reflected that breast milk alone cannot provide sufficient nutrients for infant growth and development was a misperception [47]. However, there are still a number of mothers who do not have sufficient breast milk to ensure baby’s growth. Moreover, preterm infants and infants with specific diseases need special medical formula to ensure their growth [48]. Therefore, formula is essential for these infants.

Evidence has shown that breastfeeding is associated with reduced infant morbidity and mortality attributable to diarrheal and respiratory diseases. A cohort study had demonstrated that an estimated 53% of diarrhea hospitalizations could have been prevented per month by exclusive breastfeeding and 31% by partial breastfeeding in the first 8 months after birth [49]. Another cohort study supports this result that formula feeding for ≥3 months was associated with higher odds of diarrhea between 6 and 12 months [50]. A recent systematic review showed that the pooled relative risk of diarrhea incidence among infants aged ≤ 6 months was 0.37 (95% CI: 0.27; 0.50) and was 0.46 (95% CI: 0.28; 0.78) among infants aged > 6 months [51]. Consistent with these previous studies, we found that breastfeeding was associated with a lower risk of diarrheal and respiratory diseases during the past two weeks in infants under 12 months of age. This could be due to formula-fed infants having fewer opportunities to receive antibodies and bacterial communities from their mothers [9,10,11,12,13,14,15,16,17,18,19,20,21,22,23,24,25,26,27,28,29,30,31,32,33,34,35,36,37,38,39,40,41,42,43,44,45,46,47,48,49,50,51,52].

Many studies have shown that the likelihood of anemia might increase by exclusive breastfeeding for six months and continued breastfeeding after 6 months [53,54]. This study found that breastfed infants had lower hemoglobin level than that of formula-fed infants, except for the 0–2 months group. However, infants in our study were relatively heavy and had high z-scores of lengths for age and weight for age, especially those breastfeeding infants. This was consistent with two Chinese cohort studies, which showed that breastfeeding beyond six months of age was associated with poor iron status and anemia [55]. In China, the home-made complementary foods for infants were mostly plant-based, the same as that for adults. Parents tended to provide plant-based complementary foods, such as cereal, vegetables, and fruits before animal food, which could help infants to satisfy their iron and zinc requirements [56]. Although the benefits of breastfeeding are indisputable, these findings imply that we should monitor hemoglobin level in infants with prolonged breastfeeding duration. The caregivers should introduce iron-containing complementary foods in time, such as liver paste, yolk, and meat paste, especially in infants who were exclusively breastfed.

This study has several strengths. By use of the most recent nationally representative data available, it provides an up-to-date description of breastfeeding and formula feeding among infants under one year in China. In case of multiple confound variables of infant physical development and diarrheal and respiratory diseases, we adopted a propensity score matching method to control various potential sources of biases, including sociodemographic, biomedical, behavioral, and maternal factors.

The method and data suffer from some limitations. First, although being a powerful and commonly used approach for decreasing bias in observational studies, propensity score matching has statistical limitations. Despite many covariates had been considered in our analysis, some important factors that could affect infants’ physical development were left out due to a lack of data, such as seasonal influence, vaccination information, the amount of breastmilk and formula intake, daily dietary energy intake from complementary food, mother’s lifestyle during pregnancy, and maternal dietary intake during pregnancy and during lactation. Second, due to the lack of the proportion of breastmilk and formula intake, predominated milk for the infants were unclear. Thus, the effect of mixed feeding was not considered. Third, due to the lack of information, further studies should be conducted to learn about the association between feeding patterns and other diseases, for example allergies, which are also critical health outcomes for infants.

Nonetheless, to the best of our knowledge, this study included the largest and representative infant sample in China, and may thus be useful for illustrating the relation between feeding patterns and health status among 0–11 months infants.

## 5. Conclusions

Breastfeeding and formula feeding has similar effect on infant physical development. Breastfeeding provided protection against diarrheal and respiratory illnesses. Infants aged 3–11 months who were breastfeeding had lower hemoglobin than that of formula-fed infant, indicating that they should consume more iron rich complementary foods. In conclusion, the findings of our study suggest that infants within 12 months should keep breastfeeding, and that policies are needed to achieve longer breastfeeding durations.

## Figures and Tables

**Table 1:** Infant, family, and maternal characteristics before and after matching, N (%).

Covariates	Before Matching			After Matching		
	Breastfeeding	Formula Feeding	*p*-Value	Breastfeeding	Formula Feeding	*p*-Value
N	2892	1082		941	941	
Age (day)	177.4 ± 90.4	247.6 ± 88.9	<0.001	235.7 ± 89.8	236.5 ± 89.0	0.888
Age group			<0.001			1.000
0–2 months	549 (19.0)	71(6.6)		71 (7.6)	71 (7.6)	
3–5 months	1081 (37.4)	206(19.0)		204 (20.7)	204 (20.7)	
6–8 months	716 (24.8)	290(26.8)		278 (29.5)	278 (29.5)	
9–11 months	546 (18.9)	515 (47.6)		388 (41.2)	388 (41.2)	
Sex			0.811			0.782
Boys	1469 (50.8)	545 (50.4)		472 (50.2)	478 (50.8)	
Girls	1423 (49.2)	537 (49.6)		469 (49.8)	463 (49.2)	
Area			<0.001			0.670
Big city	673 (23.3)	334 (30.9)		288 (30.6)	292 (31.0)	
Medium and small city	807 (27.9)	412 (38.1)		307 (32.6)	327 (34.8)	
Normal rural	1043 (36.1)	245 (22.6)		246 (26.1)	231 (24.5)	
Poor rural	369 (12.8)	91 (8.4)		100 (10.6)	91 (9.7)	
Annual income per capita			<0.001			0.973
Low	997 (34.5)	290 (26.8)		271 (28.8)	275 (29.2)	
Medium	851 (29.4)	284 (26.2)		247 (26.2)	242 (25.7)	
High	781 (27.0)	384 (35.5)		322 (34.2)	318 (33.8)	
Unknown	263 (9.1)	124 (11.5)		101 (10.7)	106 (11.3)	
Born to term			0.002			1.000
Term	2598 (89.8)	935 (86.4)		823 (87.5)	823 (87.5)	
Preterm	294(10.2)	147(13.6)		118(12.5)	118(12.5)	
Birth weight			<0.001			0.545
<2500 g	50(1.7)	66(6.1)		35(3.7)	44(4.7)	
2500–4000 g	2682(92.7)	970(89.6)		865(91.9)	853(90.6)	
>4000 g	160(5.5)	46(4.3)		41(4.4)	44(4.7)	
Sleep duration			0.013			0.886
Sufficient	1727(59.7)	693(64.0)		598(63.5)	595(63.2)	
Insufficient	1165(40.3)	389(36.0)		343(36.5)	346(36.8)	
Physical activity time			<0.001			0.806
High	1390 (48.1)	761 (70.3)		633 (67.3)	628 (66.7)	
Low	1502 (51.9)	321 (29.7)		308 (32.7)	313 (33.3)	
Types of complementary food	4.2 ± 2.2	3.0 ± 2.4	<0.001	3.8 ± 2.4	4.0 ± 2.2	0.201
Maternal age			0.057			0.979
<20 years old	118 (4.1)	32 (3)		30 (3.2)	29 (3.1)	
20–35 years old	2572 (88.9)	956 (88.4)		831 (88.3)	830 (88.2)	
>35 years old	202 (7.0)	94 (8.7)		80 (8.5)	82 (8.7)	
Gestational weight gain			0.560			0.872
Insufficient	784 (27.1)	278 (25.7)		231 (24.5)	236 (25.1)	
Appropriate	1051 (36.3)	391 (36.1)		345 (36.7)	351 (37.3)	
Excessive	1057 (36.5)	413 (38.2)		365 (38.8)	354 (37.6)	
Mother’s education			<0.001			0.995
Elementary school or below	258 (8.9)	74 (6.8)		68(7.2)	69 (7.3)	
Junior school	1396 (48.3)	391 (36.1)		362(38.5)	361 (38.4)	
High school	579 (20.0)	266 (24.6)		222(23.6)	218 (23.2)	
College and above	659 (22.8)	351 (32.4)		289 (30.7)	293 (31.1)	
Mother’s occupation			<0.001			0.827
Professionals	614 (21.2)	349 (32.3)		259 (27.5)	271 (28.8)	
Agricultural industry	334 (11.5)	86 (7.9)		77 (8.2)	76 (8.1)	
Others	1944 (67.2)	647 (59.8)		605 (64.3)	594 (63.1)	

**Table 2:** Infant physical development in breastfeeding and formula feeding group after matching.

	Breast Feeding	Formula Feeding
0–2 months		
weight-for-age z score	1.3 ± 1.6	1.5 ± 2.4
length-for-age z score	0.4 ± 2.1	0.7 ± 3.1
weight-for-length z score	1.1 ± 1.8	1.0 ± 1.9
3–5 months		
weight-for-age z score	1.2 ± 1.1	1.0 ± 1.6
length-for-age z score	0.7 ± 1.2	0.6 ± 1.7
weight-for-length z score	0.9 ± 1.3	0.6 ± 1.7
6–8 months		
weight-for-age z score	1.3 ± 1.2	1.1 ± 1.0
length-for-age z score	0.7 ± 1.6	0.7 ± 1.7
weight-for-length z score	0.9 ± 1.4	0.6 ± 1.4 *
9–11 months		
weight-for-age z score	1.1 ± 1.1	0.9 ± 1.3 *
length-for-age z score	0.3 ± 1.3	0.3 ± 1.3
weight-for-length z score	1.0 ± 1.3	0.7 ± 1.4 **

* *p* < 0.05; ** *p* < 0.01.

**Table 3:** Association between breastfeeding and prevalence of respiratory diseases and diarrhea in the last two weeks, N (%).

	N	Respiratory Diseases	OR (95% CI)	Diarrhea	OR (95% CI)
0–2 months					
Formula feeding	71	8 (11.3)	ref	10 (14.1)	ref
Breastfeeding	71	3 (4.2)	0.35 (0.09, 1.37)	3(4.2)	0.27 (0.07, 1.02)
3–5 months					
Formula feeding	191	38 (18.6)	ref	29 (14.2)	ref
Breastfeeding	191	23 (11.3)	0.56 (0.32, 0.97) *	23 (11.3)	0.77 (0.43, 1.38)
6–8 months					
Formula feeding	278	65 (23.4)	ref	42 (15.1)	ref
Breastfeeding	278	61 (21.9)	0.92 (0.62, 1.37)	25 (9.0)	0.56 (0.33, 0.94) *
9–11 months					
Formula feeding	388	105 (27.1)	ref	53 (13.7)	ref
Breastfeeding	388	93 (24.3)	0.85 (0.61, 1.17)	53 (13.7)	1.00 (0.66, 1.51)
Total					
Formula feeding	928	216 (23.3)	ref	134 (14.4)	ref
Breastfeeding	928	180 (19.4)	0.79 (0.64, 0.99) *	104 (11.2)	0.75 (0.57, 0.98) *

* *p* < 0.05.

## Data Availability

The data cannot be used publicly.
